# Pedometer-Based Internet-Mediated Intervention For Adults With Chronic Low Back Pain: Randomized Controlled Trial

**DOI:** 10.2196/jmir.2605

**Published:** 2013-08-22

**Authors:** Sarah L Krein, Reema Kadri, Maria Hughes, Eve A Kerr, John D Piette, Rob Holleman, Hyungjin Myra Kim, Caroline R Richardson

**Affiliations:** ^1^VA Ann Arbor Center for Clinical Management ResearchVA Ann Arbor Healthcare SystemAnn Arbor, MIUnited States; ^2^Division of General MedicineDepartment of Internal MedicineUniversity of MichiganAnn Arbor, MIUnited States; ^3^Center for Statistical Consultation and ResearchUniversity of MichiganAnn Arbor, MIUnited States; ^4^Department of Family MedicineUniversity of MichiganAnn Arbor, MIUnited States

**Keywords:** chronic pain, Internet, randomized controlled trial, exercise therapy

## Abstract

**Background:**

Chronic pain, especially back pain, is a prevalent condition that is associated with disability, poor health status, anxiety and depression, decreased quality of life, and increased health services use and costs. Current evidence suggests that exercise is an effective strategy for managing chronic pain. However, there are few clinical programs that use generally available tools and a relatively low-cost approach to help patients with chronic back pain initiate and maintain an exercise program.

**Objective:**

The objective of the study was to determine whether a pedometer-based, Internet-mediated intervention can reduce chronic back pain-related disability.

**Methods:**

A parallel group randomized controlled trial was conducted with 1:1 allocation to the intervention or usual care group. 229 veterans with nonspecific chronic back pain were recruited from one Department of Veterans Affairs (VA) health care system. Participants randomized to the intervention received an uploading pedometer and had access to a website that provided automated walking goals, feedback, motivational messages, and social support through an e-community (n=111). Usual care participants (n=118) also received the uploading pedometer but did not receive the automated feedback or have access to the website. The primary outcome was measured using the Roland Morris Disability Questionnaire (RDQ) at 6 months (secondary) and 12 months (primary) with a difference in mean scores of at least 2 considered clinically meaningful. Both a complete case and all case analysis, using linear mixed effects models, were conducted to assess differences between study groups at both time points.

**Results:**

Baseline mean RDQ scores were greater than 9 in both groups. Primary outcome data were provided by approximately 90% of intervention and usual care participants at both 6 and 12 months. At 6 months, average RDQ scores were 7.2 for intervention participants compared to 9.2 for usual care, an adjusted difference of 1.6 (95% CI 0.3-2.8, *P*=.02) for the complete case analysis and 1.2 (95% CI -0.09 to 2.5, *P*=.07) for the all case analysis. A post hoc analysis of patients with baseline RDQ scores ≥4 revealed even larger adjusted differences between groups at 6 months but at 12 months the differences were no longer statistically significant.

**Conclusions:**

Intervention participants, compared with those receiving usual care, reported a greater decrease in back pain-related disability in the 6 months following study enrollment. Between-group differences were especially prominent for patients reporting greater baseline levels of disability but did not persist over 12 months. Primarily, automated interventions may be an efficient way to assist patients with managing chronic back pain; additional support may be needed to ensure continuing improvements.

**Trial Registration:**

ClinicalTrials.gov NCT00694018; http://clinicaltrials.gov/ct2/show/NCT00694018 (Archived by WebCite at http://www.webcitation.org/6IsG4Y90E).

## Introduction

Low back pain is a significant health problem with approximately one-half of adults reporting back pain during a given year [1-3]. Low back pain that persists for longer than 3 months is considered chronic [4], and the longer the pain persists the greater the risk for long-term disability [5]. Chronic back pain is associated with functional limitations, social isolation, unemployment, and lost productivity [5-7], making it one of the most costly health conditions in the United States [8-11].

Exercise therapy has proven benefits for managing chronic back pain [12-14]. Specifically, exercise can prevent recurrence, reduce pain, improve function, and decrease disability for patients with chronic back pain [12,13,15-19]. It is also generally recognized that, to be effective, patients have to be willing and able to perform the recommended exercise and for continuing benefits remain adherent to the exercise program [18,20,21]. However, there are few efficient and effective strategies to help patients engage in exercise therapy for managing their chronic low back pain.

Internet-based programs are an increasingly popular option for promoting healthy behaviors, such as those related to diet and exercise, and for delivering behavior change interventions [22-24]. Studies have shown that the Internet can be used to successfully promote weight loss [25], increase physical activity [26], and improve patient self-activation [27] or self-management behaviors [22]. Studies of Internet-based interventions for pain, while somewhat limited, show a generally positive effect on pain levels and, to some extent, activity [27-30]. No studies, however, have focused primarily on exercise to reduce pain-related disability and improve patient function.

We conducted a randomized trial to investigate whether a pedometer-based, Internet-mediated intervention designed to assist patients with initiating and maintaining a regular walking program would reduce pain-related disability and functional interference among patients with chronic back pain at 6 months and over a 12-month timeframe.

## Methods

### Design Overview

We conducted a parallel group randomized controlled trial with participants allocated in a 1:1 ratio to the intervention or enhanced usual care (NCT00694018). This research was approved by the Department of Veterans Affairs (VA), Ann Arbor Healthcare System institutional review board. The study protocol, including conceptual framework, is described in detail elsewhere [31], with key elements summarized below. There were no significant changes in methods following study initiation.

### Setting and Participants

Participants were recruited from one VA Healthcare System between May 2009 and March 2011. Eligible participants were aged 18 years or older and identified through provider referrals to back class and use of the VA electronic medical record system. Specifically, we identified patients who had 2 or more outpatient encounters in the previous 12 months with a diagnosis of back pain with no neurologic findings (ICD-9-CM codes 724.2, 724.5, 846.0-846.9).

Study staff used a standardized protocol to screen potential participants by phone or, for a minority of patients who could not be reached by phone, in person when they arrived for back class. Eligibility criteria included: (1) persistent back pain >3 months, (2) self-reported sedentary lifestyle (defined as <150 minutes of physical activity per week in accordance with the US Department of Health and Human Services 2008 Physical Activity Guidelines for Americans [32]), (3) weekly access to a computer with a USB port and Internet access, (4) ability to provide written informed consent and communicate in English, (5) community residence, (6) ability to walk at least one block, and (7) report they are not pregnant. Prior to participation, all eligible patients had to attend back class and obtain medical clearance. Back class, led by a physical therapist, provided general education about managing back pain. Participants also performed back-specific strengthening and stretching exercises under the supervision of a physical therapist.

Eligible participants then attended a study enrollment session at which time they provided written informed consent and were told they were helping test an Internet-based program and would be assigned to one of two groups: (1) an enhanced care group that would upload pedometer data weekly and have access to a study website and computer discussion group (Internet support group), or (2) a usual care group that would upload pedometer data monthly (monthly upload group). All participants received an uploading pedometer (the Omron HJ-720ITC, which stores 42 days of step-count data and has an embedded USB port [33]), along with general guidance on using the pedometer and instructions for logging onto and uploading data to the study website. To establish a baseline step count that was not influenced by use of the pedometer information, participants were instructed to wear their pedometer for 7 days with the display covered before completing their first upload.

### Randomization

After completing the baseline survey, uploading 7 days of useable pedometer data, and receiving medical clearance, each participant was randomly allocated in a 1:1 ratio to the intervention or usual care group by a computer program (using a random number generator). The program also generated an email message to inform participants about their group assignment (Internet support or monthly upload) and instructions to remove the sticker covering the pedometer display.

### Intervention

The study intervention, based on the Stepping Up to Health program [31,34], consisted of three primary components: (1) the uploading pedometer, (2) a website that provided automated goal setting and feedback, targeted messages, and educational materials, and (3) an e-community [31] (see Multimedia Appendix 1). The conceptual framework and more detailed description of the intervention components are published elsewhere [31]. Briefly, participants were instructed to wear their pedometer from the time they got up in the morning until they went to bed. Intervention participants then received weekly email reminders to upload their pedometer data, which was used to establish weekly individualized walking goals. Each participant’s goal was based on their average total step count in the prior week with a fixed number of steps (800) added to promote a gradual increase in walking for the following week. The step count goal was emailed to the participant each week and posted on the study website.

The study website, which was fully accessible to intervention participants, also included graphical and written feedback about their progress toward their walking goals and contained pain- or activity-related motivational and informational messages. These messages included quick tips, which changed every other day, and weekly updates about topics in the news. Back class materials, which included handouts about topics such as body mechanics, use of cold packs, lumbar rolls, and good posture, as well as a video demonstrating specific strengthening and stretching exercises were also available on the website. Finally, the website based e-community or forum allowed participants to post suggestions, ask questions, and share stories. Topics discussed included mental health concerns, such as depression, strategies for walking such as walking the dog or interesting hiking trails, walking during hot weather and cold weather, and use of alternative pain management strategies such as massage. Research staff participated in and monitored the forum posts as well as used the forum as a venue to generate competitions to encourage meeting walking goals.

### Enhanced Usual Care

Usual care participants also received the uploading pedometer and monthly email reminders to upload their pedometer data. However, they did not receive any goals or feedback and their access to the study website was limited to completing surveys and reporting adverse events only.

### Monitoring of Adverse Events

Both groups were encouraged to report any health problems via the website, email, or phone. Four weeks after randomization and every 8 weeks thereafter, participants were prompted to complete a survey that asked about specific adverse events (eg, heart attack) and symptoms such as shortness of breath. This information was closely monitored and participants with potentially serious health-related problems were contacted for further assessment and follow-up.

### Outcomes and Follow-Up

Outcomes were measured at baseline, 6 months, and 12 months using a survey administered through the study website, or by a mailed questionnaire if the participant could not complete the computerized instrument. The prespecified primary outcome was pain-related disability at 12 months, as measured using the back pain-specific Roland Morris Disability Questionnaire (RDQ) [35], and a generic pain-related function measure from the Medical Outcomes Study (MOS) [36]. The RDQ, a 24-item scale with higher scores indicating greater disability, has been widely used in back pain studies as a measure of self-perceived disability [35,37-39]. The MOS measure assesses the effect of pain on mood and behaviors as well as pain severity, with higher scores also indicating greater functional interference [36].

Pain intensity, a secondary outcome, was evaluated using a numeric rating scale with standard anchors (0=“no pain” and 10=“worst pain imaginable”) [40]. Walking, also a secondary outcome, was measured as the average number of steps per day over the past 7 days using step-count data collected through the pedometer uploads. Other secondary outcomes included pain-related fear-avoidance, measured using the Fear-Avoidance Beliefs Questionnaire physical activity subscale (higher scores reflect higher levels of fear-avoidance) [41], and self-efficacy for exercise, measured using the Exercise Regularly Scale, with higher scores indicating higher levels of self-efficacy [42]. Additional data collected at baseline included age, gender, race, employment status, education level, relationship status, average household income, body mass index, and use of narcotic medications for pain management. An administrative interface to the website provided data on the number of pedometer uploads and website log-ins.

### Sample Size

Sample size was based on the RDQ score as the primary outcome with a minimally detectable and clinically meaningful effect size determined as a difference of 0.4 standard deviation (SD) in change scores or a 2-point difference, based on published data [38,43,44]. To detect a difference of 0.4 SD with 80% power using a two-sided 0.05 level 2 group *t* test, we sought to enroll 130 subjects in each group, to allow for an attrition rate of 25% at 1 year.

### Statistical Analysis

The analyst assessing final trial outcomes was blinded to study assignment. All analyses were conducted using an intent-to-treat approach with participants analyzed according to original group assignment. We conducted both complete and all case analyses to assess differences between groups in change in RDQ at 6 and 12 months. The complete case analysis was conducted using multiple linear regression models with adjustment for baseline values of the RDQ. The all case analysis was conducted using linear mixed-effects models, allowing us to use data from all participants and provide an unbiased estimate of the outcome, assuming data are missing at random [45]. For example, for our 12-month analysis, RDQ scores at baseline and 12 months were used as dependent variables, with the primary independent variables consisting of an indicator for the intervention group and an interaction term of time by intervention group. Each participant’s data was modeled using a random intercept to allow within-patient correlation of the repeated measures. Adjustment for covariates was only planned if an imbalance was found between groups at baseline.

We also conducted a post hoc subgroup analysis of participants with baseline RDQ scores of ≥4. As a pragmatic trial we did not screen based on RDQ scores, and some participants had baseline scores that were very low or even 0. Thus, to assess the effect of the intervention on participants reporting at least modest levels of back pain-related disability at baseline, we conducted a subgroup analysis of those with baseline RDQ scores of ≥4 using the same methods previously described.

Analyses were conducted using Stata 11.2 and all reported *P* values are from adjusted analyses.

## Results

### Summary

**Figure 1:**
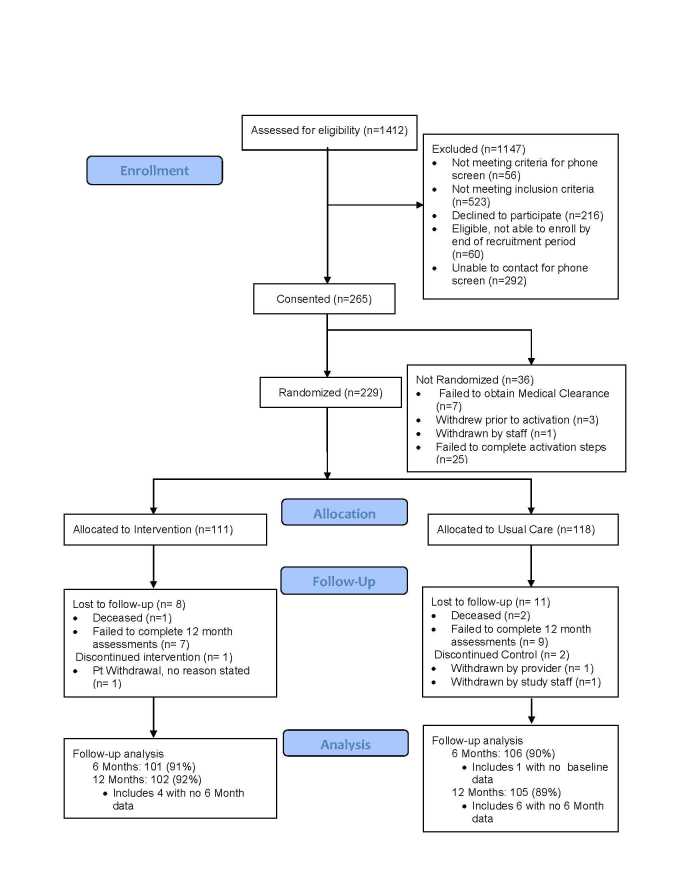
Study flow diagram.

Over 1400 potential participants (Figure 1) were assessed for eligibility. Primary reasons for ineligibility were lack of regular access to a computer or the Internet (n=310) and being too physically active (n=159). Of those determined to be eligible, 229 completed all of the steps in the enrollment process, with 111 randomly allocated to the Internet-mediated intervention and 118 to enhanced usual care. Primary outcome data were provided by 91% of intervention and 90% of usual care participants at 6 months, and by 92% of those in the intervention group and 89% receiving usual care at 12 months.

### Baseline Characteristics

Participants were predominantly male and white, with an average age of 51 years (Table 1). The majority had completed some college, were either married or living with someone as a couple, and the mean body mass index was over 30. At baseline, less than 40% of participants reported being employed full- or part-time and over 40% reported taking narcotic medications for their back pain. None of the observed differences in baseline characteristics were statistically significant.

### Primary Outcomes

**Figure 2:**
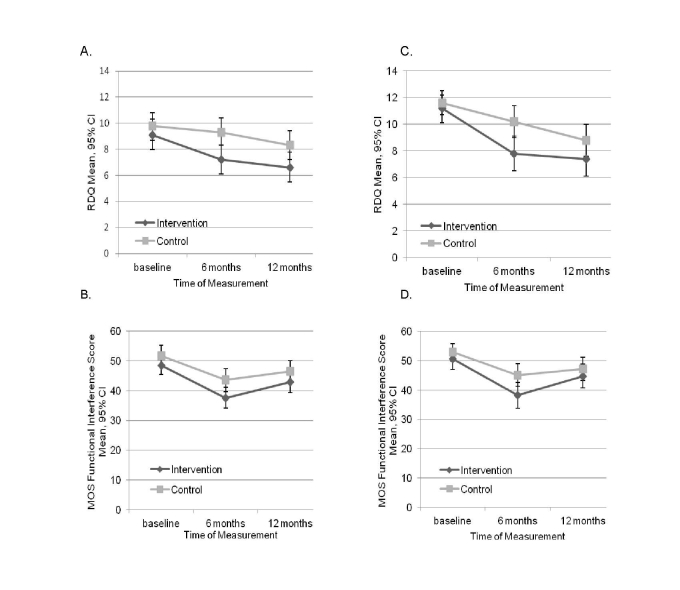
Mean RDQ scores (top) and MOS functional interference scores (bottom). A and B: full sample, C and D: patients with baseline RDQ scores ≥ 4.

At baseline, mean RDQ scores were greater than 9 in both groups (Table 1), indicating moderately severe back pain-related disability. The mean RDQ score at 6 months was 7.2 for intervention participants compared to 9.2 for those in usual care (Figure 2), an adjusted difference of 1.6 (95% CI 0.3-2.8, *P*=.02) for the complete case analysis and 1.2 (95% CI -0.09 to 2.5, *P*=.07) for the all case analysis (Table 2). When restricted to the subgroup with at least moderate back pain at baseline (RDQ score ≥4) (Figure 2, Table 2), patients in the intervention had a significant improvement in back pain-related disability compared to the control group, an adjusted difference of approximately 2 in both the complete (1.9, 95% CI 0.5-3.3, *P*=.01) and all case (1.7, 95% CI 0.3-3.0, *P*=.02) analyses. RDQ scores continued to decline between 6 and 12 months in both groups and, while scores for the intervention group remained lower than for usual care, at 12 months these differences were no longer statistically significant. The MOS function measure also suggested greater improvements in function for intervention compared to usual care participants at 6 months (Figure 2), but none of the adjusted differences were statistically significantly different.

### Secondary Outcomes

At baseline, pain severity was rated at approximately 6 on a 0-10 scale by both intervention and usual care participants (Table 1). Reported pain levels decreased in both groups at 6 months and remained lower than baseline at 12 months. The greatest change occurred between baseline and 6 months among those in the intervention group (6.0-4.7 vs 6.1-5.2 in the control group), although the adjusted difference between arms of 0.5 was not significant (Table 3).

Average step counts of slightly more than 4000 steps per day at baseline in each group increased at 6 months for intervention patients, with an adjusted difference between groups of more than 700 steps. By 12 months, however, the adjusted difference between groups was only 100-200 steps. Exercise self-efficacy scores appeared to be the same or lower (worse) for both groups at 6 months, although the decrease was significantly less for those in the intervention compared to the control group, an adjusted difference of 0.8 (95% CI 0.24-1.4, *P*=.01) in the complete case analysis and 0.7 (95% CI 0.12-1.2, *P*=.02) for the all case analysis (Table 3). This difference did not persist at 12 months. There was no difference between groups in the physical activity fear-avoidance scale at any time point.

### Intervention Engagement

Intervention participants uploaded pedometer data at least once per week for a median of 32 weeks (62% of the recommended time), although more than 25% of participants uploaded data for at least 42 weeks (80% compliance). However, intervention participants logged into the website at least once per week for a median of only 20 weeks (38% of the recommended time), with approximately 20% logging in for at least 42 weeks.

### Adverse Events

During the study, approximately 600 adverse events were reported by participants (250 by those in usual care and nearly 350 by those in the intervention). These events ranged from calluses to chest pain. Worsening back pain, the most frequently reported event, accounted for 29% of events reported by the usual care group and 25% of those reported by the intervention group. Overall, more musculoskeletal events (n=112) were reported than cardiovascular events (n=85), and musculoskeletal injuries were more likely to be reported by participants in the intervention group compared to those in usual care. However, no major study-related adverse events (eg, heart attack) were identified for either group.

**Table 1:** Participant baseline characteristics.

Characteristic		Internet-mediated intervention (n=111)	Enhanced usual care (n=118)
Age (y), mean (SD)		51.2 (12.5)	51.9 (12.8)
Male (%)		89	86
**Race (%)**			
	White	74	86
	Black	13	9
	Other or prefer not to answer	14	5
**Education level (%)**			
	High school or less	29	25
	Some college	56	59
	4 years of college or more	16	16
Married or living with a partner (%)		59	68
Employed full-time or part-time (%)		39	31
**Annual household income (%)**			
	<US $10,000	18	13
	US $10,000-$39,999	61	54
	≥ US $40,000	21	33
Take narcotic medications for back pain (%)		41	49
General health status, fair or poor (%)		41	43
Body mass index, mean (SD)		30.6 (5.7)	31.6 (5.5)
RDQ score (0-24)^a^, mean (SD)		9.1 (6.0)	9.8 (5.7)
MOS pain-related functional interference score (0-100)^a^, mean (SD)		48.5 (18.6)	51.8 (16.3)
Level of pain severity, 0-10 scale^a^, mean (SD)		6.0 (1.9)	6.1 (1.6)
Daily step counts, mean (SD)		4492.9 (2749.9)	4321.9 (2285.4)
Exercise self-efficacy score, 1-10^b^, mean (SD)		6.8 (2.1)	6.5 (2.3)
Physical activity fear-avoidance behavior scale, 0-28^a^, mean (SD)		13.9 (5.9)	15.1 (6.0)

^a^lower scores are better

^b^higher scores are better

**Table 2:** Primary outcomes, back pain-specific and general pain-related function.

Primary outcome				Adjusted between-group difference^a^ (95% CI)			
			Complete case		*P* value	All case	*P* value
**RDQ score (0-24)**							
	6 months		1.6 (0.3 to 2.8)		.02	1.2 (-0.09 to 2.5)	.07
	12 months		1.2 (-0.3 to 2.7)		.11	0.7 (-0.8 to 2.2)	.38
**MOS pain-related functional interference score (0-100)**							
	6 months		3.6 (-0.51 to 7.7)		.09	2.5 (-1.5 to 6.5)	.23
	12 months		0.1 (-4.0 to 4.2)		.97	-1.4 (-5.4 to 2.5)	.48
**Subgroup with RDQ scores** ≥**4 at baseline RDQ score (0-24)**							
	6 months		1.9 (0.5 to 3.3)		.01	1.7 (0.3 to 3.0)	.02
	12 months		1.1 (-0.6 to 2.7)		.20	0.8 (-0.8 to 2.4)	.34
**MOS pain-related functional interference score (0-100)**							
	6 months		4.6 (-0.1 to 9.3)		.05	3.8 (-0.7 to 8.3)	.10
	12 months		-0.5 (-5.0 to 4.0)		.83	-1.5 (-5.8 to 2.8)	.49

^a^Adjusted for baseline values and calculated as pain or function in enhanced usual care group minus Internet-mediated intervention group so that positive scores reflect greater improvement in the intervention group.

**Table 3:** Secondary outcomes.

Secondary outcome		Mean (SD)		Adjusted between-group difference^a^ (95% CI)			
		Internet-mediated intervention	Enhanced usual care	Complete case	*P* value	All case	*P* value
**Level of pain severity, 0-10 scale** ^b^							
	6 months	4.7 (2.1)	5.2 (2.1)	0.5 (-0.01 to 0.98)	.06	0.5 (-0.03 to 0.9)	.07
	12 months	5.4 (2.2)	5.6 (2.0)	0.1 (-0.4 to 0.5)	.81	0.04 (-0.4 to 0.5)	.86
**Daily step counts** ^c,d^							
	6 months	5370.0 (3180.8)	4682.5 (2925.0)	725.5 (-193.6 to 1644.7)	.12	724.0 (-75.2 to 1523.2)	.08
	12 months	4681.8 (3000.6)	4758.1 (2991.1)	122.4 (-623.9 to 868.6)	.75	143.4 (-460.2 to 747.1)	.64
**Exercise self-efficacy score** ^c^							
	6 months	6.7 (2.4)	5.7 (2.5)	0.8 (0.24 to 1.4)	.01	0.7 (0.12 to 1.2)	.02
	12 months	6.4 (2.6)	5.9 (2.3)	0.3 (-0.3 to 0.9)	.32	0.2 (-0.4 to 0.74)	.55
**Physical activity fear-avoidance behavior scale** ^b^							
	6 months	13.2 (6.0)	14.0 (5.9)	0.6 (-0.88 to 2.1)	.42	-0.1 (-1.6 to 1.5)	.94
	12 months	13.3 (6.7)	15.1 (6.1)	1.1 (-0.5 to 2.7)	.18	0.6 (-1.1 to 2.2)	.50

^a^Adjusted for baseline values and calculated as pain or function in enhanced usual care group minus Internet-mediated intervention group so that positive scores reflect greater improvement in the intervention group.

^b^lower scores are better

^c^higher scores are better

^d^Pedometer data: intervention (n=84 at 6 months, n=78 at 12 months), usual care (n=70 at 6 months, n=68 at 12 months).

## Discussion

### Principal Findings

Improving management of chronic pain is a significant public health challenge and moral imperative according to a recent Institute of Medicine report [8]. More than 1 million adults in the United States have chronic pain, with low back pain being the most frequently reported condition [8]. Our findings show that an automated, Internet-mediated walking intervention may help to reduce back pain-related disability among patients with chronic back pain, although the benefits did not persist for the entire 12-month study period. Improvement was greatest for those individuals reporting moderate to severe levels of pain-related disability at baseline.

The functional results observed are generally similar to those found in other recent studies of non-invasive interventions, such as yoga and massage [46,47]. These studies also tend to show more rapid improvements for those receiving the intervention but with gradual improvements over time for those in usual care. Moreover, if we employ the criteria proposed by Jordan and colleagues [48] to classify patients as clinically improved or at least possibly improved (compared with not improved), as defined by a reduction in the RDQ score of at least 30% at 6 months, we find that 46% of those in the intervention versus 27% in the control group would meet this definition. Although we did not have a global health question and so are unable to isolate what proportion would qualify as definitely improved, this classification generally corresponds with other measures that suggest clinical improvement, such as return to work, less pain, improved function, and fewer physician visits [48]. Thus, we believe that our findings suggest that automated, remotely delivered interventions can be effectively used to promote a more rapid reduction in back pain-related disability and supplement care for patients with chronic low back pain. Further investigation is needed, however, to understand the characteristics of patients who had an early or enduring response to the intervention so that we may better target patients most likely to benefit and broaden the response.

Given the proven benefits of exercise for managing low back pain [19], a key component of the intervention focused on increasing daily step counts (ie, walking). During the first 6 months of the study, we saw an increase of nearly 700 more steps or one-third of a mile per day among intervention compared to usual care participants. Although not a statistically significant difference, we believe that even modest increases in activity can be beneficial. As one intervention participant noted: “I didn’t know what the walking could do for me. But...it seemed to alleviate my back pain...the true test came when I had to go off the program because of my illness and the back pain returned. In fact, just up until recently when I had resumed walking.” On the other hand, step-count improvements were not sustained for the entire 12 months and poor adherence or declining engagement, as demonstrated by the percentage of patients who uploaded or logged into the website, could in part account for the lack of sustained benefit or added improvement over time. Although we do not know specific reasons for this lack of participation, these data suggest that additional strategies to keep people active and engaged may be needed. This could include, for example, an online coaching component, which has been shown to improve adherence to other types of behavioral changes [47-49].

Our monitoring of adverse events showed a higher number of reported events by intervention participants. This information was, however, collected solely through self-report and we expect that some of the difference in the overall number of events reported between groups could be due to our more frequent contact with intervention participants via email and through the website. In addition, despite the higher level of musculoskeletal events reported by intervention participants, we found no evidence that the intervention led to excessive harms. Thus, even though more work to understand the circumstances for those reporting musculoskeletal problems or worsening back pain may be required, these findings add to the evidence base to support walking as a generally safe and potentially effective intervention for some patients with chronic low back pain [49-52].

Other potential mechanisms of action are less clear. Despite a marginally greater decrease in pain levels among intervention participants at 6 months, this effect did not persist at 12 months. In addition, while there was a significant difference between groups in self-efficacy for exercise at 6 months, rather than the hypothesized improvement for those in the intervention, both groups reported lower levels of self-efficacy. However, the decline was smaller for those receiving the intervention. The reason for the decrease is not entirely clear but may be largely due to an unrealistic assessment of self-efficacy at baseline [53].

### Limitations

Among the strengths of our study are the high rate of participant follow-up and our collection of detailed adverse event information. This study also has several limitations. First, patients were recruited from only 1 medical center and the sample was predominantly male. Although more than 10% of participants were female, which is relatively high for studies using a general VA patient population, the number is not sufficient for a formal subgroup analysis. However, based on trials of similar types of interventions, we expect this approach could be even more effective among women [54]. Second, we are not able to directly compare our results to other types of back pain interventions (eg, yoga), although as previously noted the general trajectory of our primary outcome (RDQ score) appears consistent with recent trials in this area. Third, although a consistent data collection format is generally recommended [55], we used both Internet-based and paper surveys. However, prior research has demonstrated similar psychometric properties between Internet and paper-and-pencil questionnaires [55] and specifically equivalence for our primary outcome [56]. We also believe that using both modes helped to ensure a high follow-up rate. Finally, as a multifaceted intervention, we are not able to determine which elements were most effective and can only draw conclusions about the program as a whole. Nonetheless, our results highlight the importance of providing active support (eg, goal setting and feedback) to encourage walking as compared with simply giving someone a pedometer to track step counts.

### Conclusions

In sum, our findings indicate that a facilitated walking intervention that uses an uploading pedometer and the Internet may help to reduce back pain-related disability among patients with chronic back pain, at least in the short term. Additional support, however, is likely needed to ensure continuing improvements long term. Nevertheless, this type of primarily automated intervention can be used to deliver care with broad reach and could be an efficient way of delivering or supplementing care provided through traditional facility-based programs.

## Multimedia Appendix 1

Intervention components.

## Multimedia Appendix 2

CONSORT-EHEALTH checklist V1.6.2 [57].

## Abbreviations

MOS: Medical Outcomes Study
RDQ: Roland Morris Disability Questionnaire
SD: standard deviation
VA: Department of Veterans Affairs
