# Supplementary material for: Pedometer-Based Internet-Mediated Intervention For Adults With Chronic Low Back Pain: Randomized Controlled Trial
Source: J Med Internet Res. 2013 Aug 22;15(8):e181. doi: 10.2196/jmir.2605 (PMC3758050; doi:10.2196/jmir.2605)

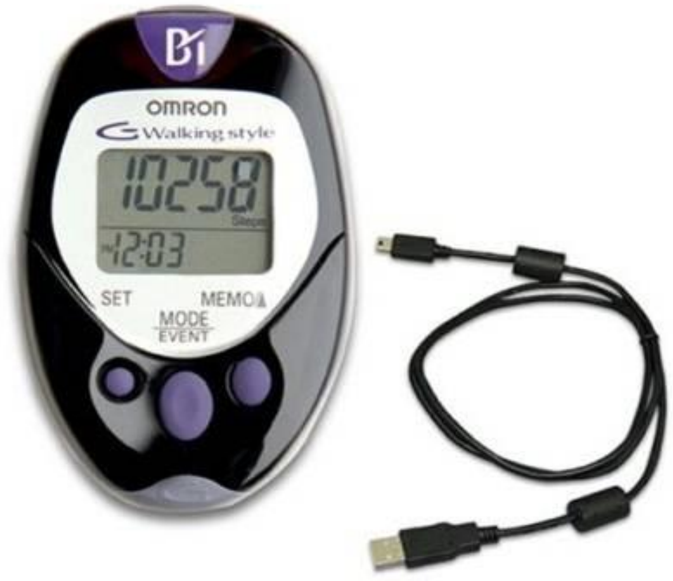

Uploading pedometer (left)

Screenshot displaying a pedometer upload (below)

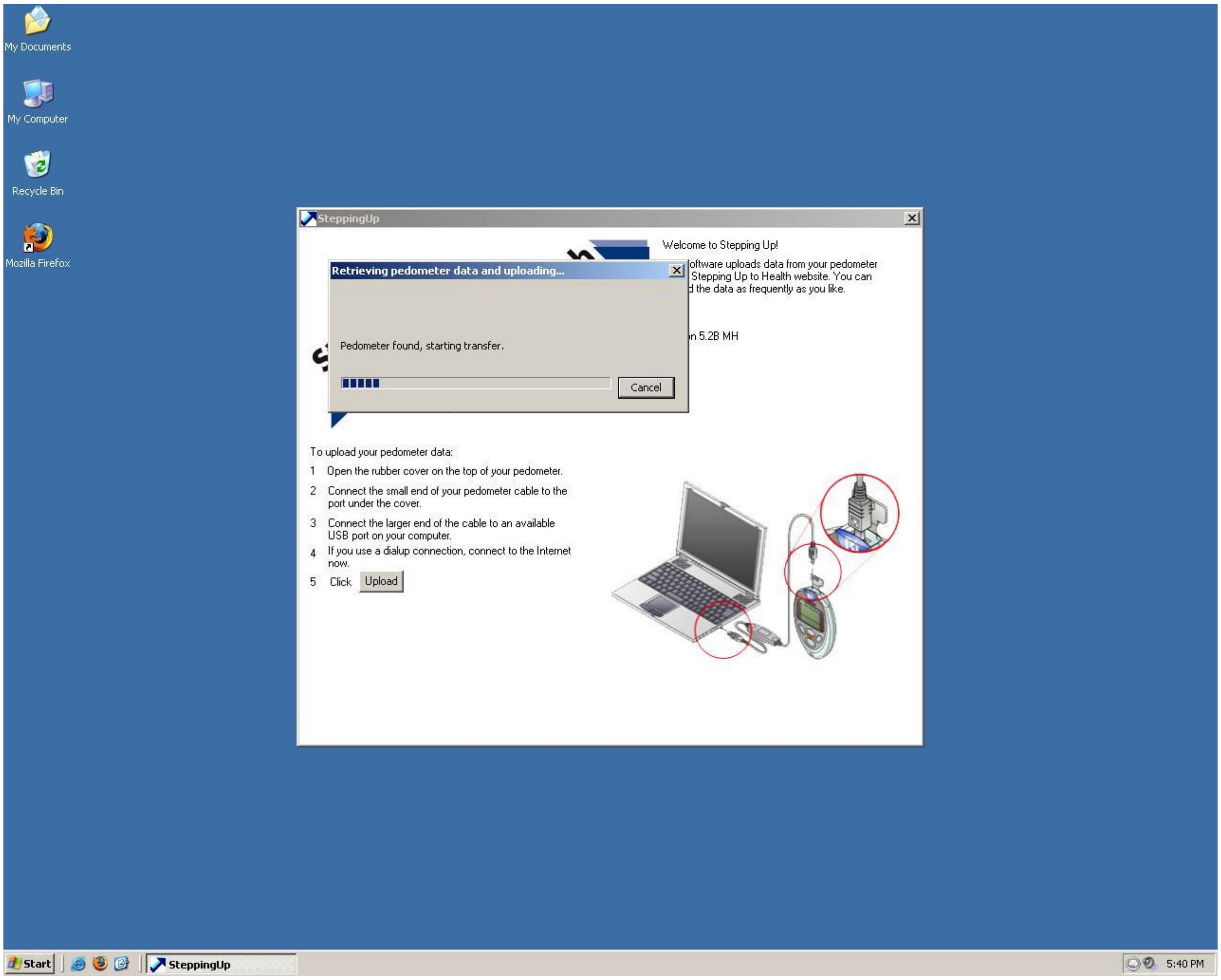

Screenshot of home page after log-in showing step count goal, feedback and active forum conversations

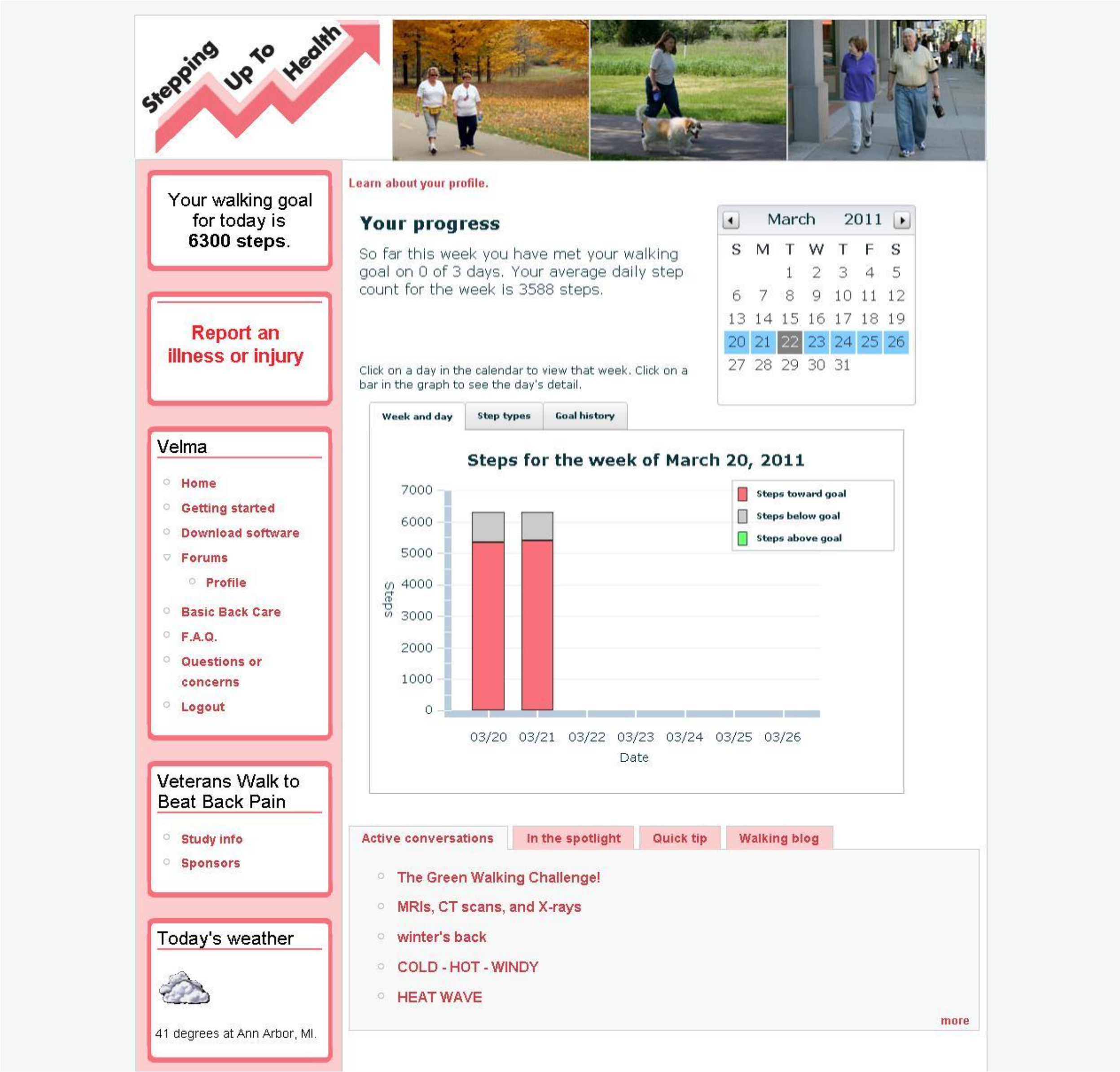

Example of In the Spotlight feature

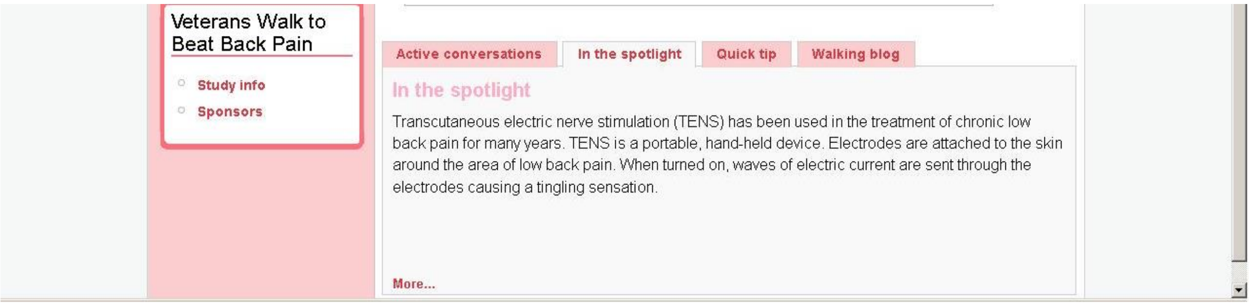

Veterans Walk to Beat Back Pain

- Study info
- Sponsors

Active conversations | In the spotlight | Quick tip | Walking blog

In the spotlight

Transcutaneous electric nerve stimulation (TENS) has been used in the treatment of chronic low back pain for many years. TENS is a portable, hand-held device. Electrodes are attached to the skin around the area of low back pain. When turned on, waves of electric current are sent through the electrodes causing a tingling sensation.

More...

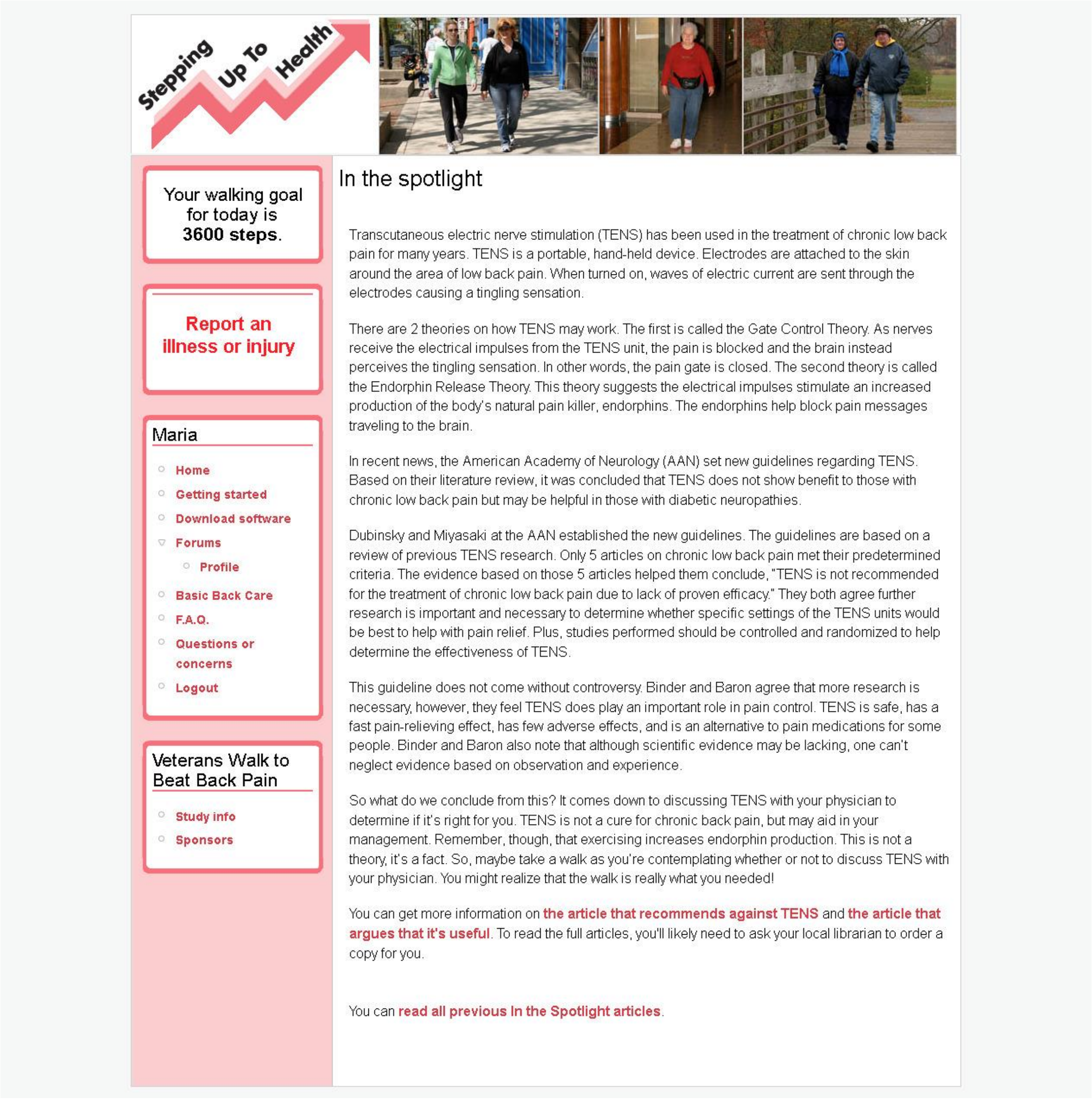

Stepping Up To Health

Your walking goal for today is **3600 steps**.

Report an illness or injury

Maria

- Home
- Getting started
- Download software
- Forums
  - Profile
- Basic Back Care
- F.A.Q.
- Questions or concerns
- Logout

Veterans Walk to Beat Back Pain

- Study info
- Sponsors

In the spotlight

Transcutaneous electric nerve stimulation (TENS) has been used in the treatment of chronic low back pain for many years. TENS is a portable, hand-held device. Electrodes are attached to the skin around the area of low back pain. When turned on, waves of electric current are sent through the electrodes causing a tingling sensation.

There are 2 theories on how TENS may work. The first is called the Gate Control Theory. As nerves receive the electrical impulses from the TENS unit, the pain is blocked and the brain instead perceives the tingling sensation. In other words, the pain gate is closed. The second theory is called the Endorphin Release Theory. This theory suggests the electrical impulses stimulate an increased production of the body's natural pain killer, endorphins. The endorphins help block pain messages traveling to the brain.

In recent news, the American Academy of Neurology (AAN) set new guidelines regarding TENS. Based on their literature review, it was concluded that TENS does not show benefit to those with chronic low back pain but may be helpful in those with diabetic neuropathies.

Dubinsky and Miyasaki at the AAN established the new guidelines. The guidelines are based on a review of previous TENS research. Only 5 articles on chronic low back pain met their predetermined criteria. The evidence based on those 5 articles helped them conclude, "TENS is not recommended for the treatment of chronic low back pain due to lack of proven efficacy." They both agree further research is important and necessary to determine whether specific settings of the TENS units would be best to help with pain relief. Plus, studies performed should be controlled and randomized to help determine the effectiveness of TENS.

This guideline does not come without controversy. Binder and Baron agree that more research is necessary, however, they feel TENS does play an important role in pain control. TENS is safe, has a fast pain-relieving effect, has few adverse effects, and is an alternative to pain medications for some people. Binder and Baron also note that although scientific evidence may be lacking, one can't neglect evidence based on observation and experience.

So what do we conclude from this? It comes down to discussing TENS with your physician to determine if it's right for you. TENS is not a cure for chronic back pain, but may aid in your management. Remember, though, that exercising increases endorphin production. This is not a theory, it's a fact. So, maybe take a walk as you're contemplating whether or not to discuss TENS with your physician. You might realize that the walk is really what you needed!

You can get more information on **the article that recommends against TENS** and **the article that argues that it's useful**. To read the full articles, you'll likely need to ask your local librarian to order a copy for you.

You can **read all previous In the Spotlight articles**.

Example of Quick Tip

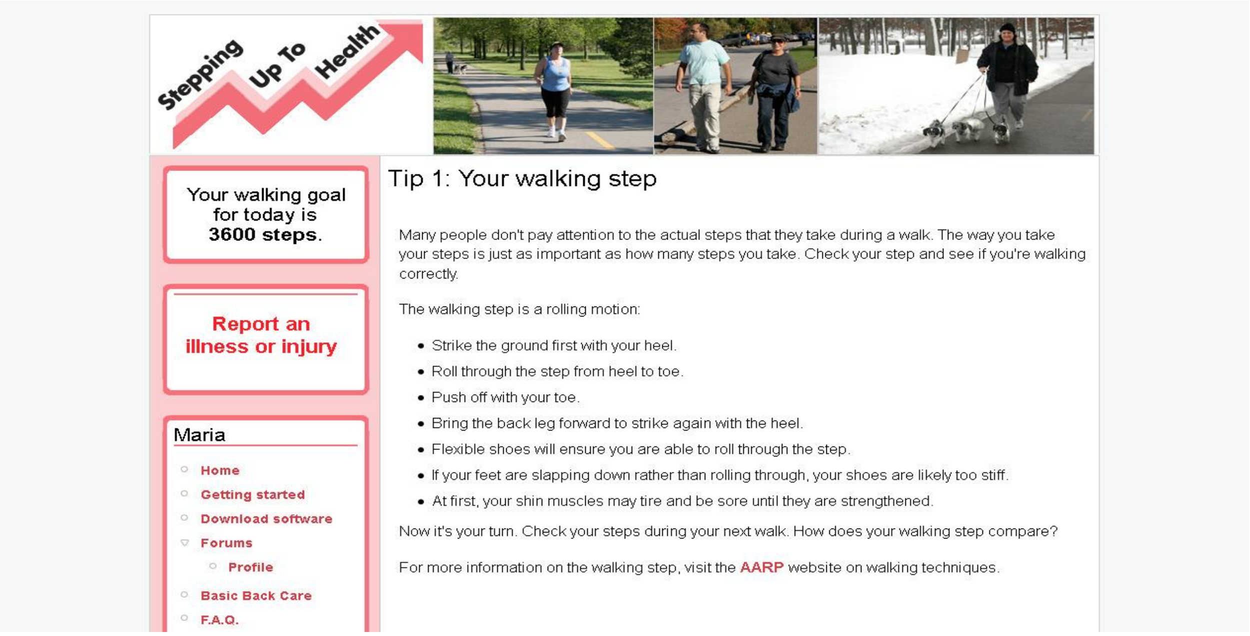

Screenshot of Back Class refresher page, with back exercise video and class handouts

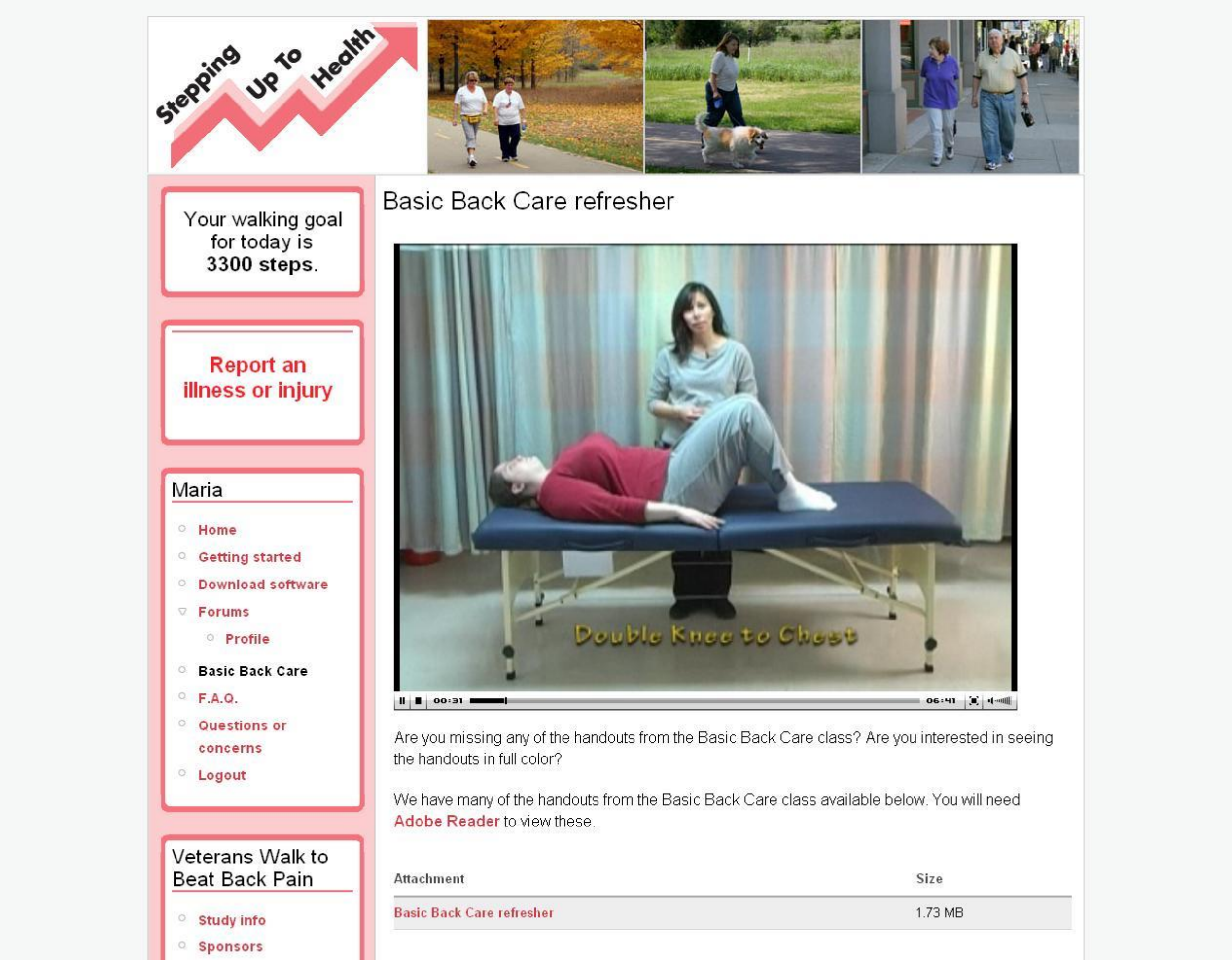

Supplement: Supplementary file 1 [file jmir_v15i8e181_app1.pdf]
