# Supplementary material for: Pedometer-Based Internet-Mediated Intervention For Adults With Chronic Low Back Pain: Randomized Controlled Trial
Source: J Med Internet Res. 2013 Aug 22;15(8):e181. doi: 10.2196/jmir.2605 (PMC3758050; doi:10.2196/jmir.2605)
Supplement: Supplementary file 2 [file jmir_v15i8e181_app2.pdf]

CONSORT-EHEALTH Checklist V1.6.2 Report

(based on CONSORT-EHEALTH V1.6), available at [http://tinyurl.com/consort-ehealth-v1-6].

2605

Date completed

2/22/2013 11:48:11

by

Krein

Pedometer-based Internet-mediated intervention for adults with chronic low back pain: A randomized controlled trial

TITLE

1a-i) Identify the mode of delivery in the title

"Internet-mediated intervention"

1a-ii) Non-web-based components or important co-interventions in title

"Pedometer-based"

1a-iii) Primary condition or target group in the title

"adults with chronic low back pain"

ABSTRACT

1b-i) Key features/functionalities/components of the intervention and comparator in the METHODS section of the ABSTRACT

"Participants randomized to the intervention received an enhanced (uploading) pedometer and had access to a website that provided automated walking goals, feedback, motivational messages and social support through an e-community (n = 111). Usual care participants (n = 118) also received the enhanced pedometer but did not receive the automated feedback or have access to the website."

1b-ii) Level of human involvement in the METHODS section of the ABSTRACT

Not reported in abstract. The intervention was mostly automated but human/staff involvement, such as posting on e-community forum and follow-up of reported potential adverse events, is described in the methods section of the manuscript.

1b-iii) Open vs. closed, web-based (self-assessment) vs. face-to-face assessments in the METHODS section of the ABSTRACT

"229 Veterans with non-specific chronic back pain were recruited from one Department of Veterans Affairs (VA) healthcare system."

"Outcomes, including the primary outcome, the Roland Morris Disability Questionnaire (RDQ), were measured at baseline, 6 months and 12 months using a survey administered through the study website."

1b-iv) RESULTS section in abstract must contain use data

"Primary outcome data were provided by approximately 90% of intervention and usual care participants at both 6 and 12 months."

Number of enrolled participants in each group is described in the methods section of the abstract:

"Participants randomized to the intervention received an enhanced (uploading) pedometer and had access to a website that provided automated walking goals, feedback, motivational messages and social support through an e-community (n = 111). Usual care participants (n = 118) . . ."

1b-v) CONCLUSIONS/DISCUSSION in abstract for negative trials

Although there were no differences at 12 months (the primary endpoint for the trial), the interim 6 months results were generally positive.

So, while not addressed in the abstract, information about uptake is included in the manuscript.

INTRODUCTION

2a-i) Problem and the type of system/solution

Pg. 5 "Exercise therapy has proven benefits for managing chronic back pain [12-14]. Specifically, exercise and staying active can prevent recurrence, reduce pain, improve function, and decrease disability for patients with chronic back pain [12, 13, 15-19]. However, there are few efficient and effective strategies to help patients engage in exercise therapy for managing their chronic low back pain."

2a-ii) Scientific background, rationale: What is known about the (type of) system

Pg 5. "Internet-based programs are an increasingly popular option for promoting healthy behaviors, such as those related to diet and exercise, and for delivering behavior change interventions [20-22]. Studies have shown that the Internet can be used to successfully promote weight loss [23], increase physical activity [24] and improve patient self activation [25] or self-management behaviors [20]. Yet, only a few studies have focused specifically on patients with chronic pain [25, 26] and none have focused primarily on exercise to reduce pain-related disability and improve patient function."

METHODS

3a) CONSORT: Description of trial design (such as parallel, factorial) including allocation ratio

Pg 5. "We conducted a randomized trial to investigate whether a pedometer-based, Internet-mediated intervention designed to assist patients with initiating and maintaining a regular walking program, would reduce pain-related disability and functional interference among patients with chronic back pain at 6 months and over a 12-month timeframe."

3b) CONSORT: Important changes to methods after trial commencement (such as eligibility criteria), with reasons

Pg 6. "There were no significant changes in methods following study initiation."

3b-i) Bug fixes, Downtimes, Content Changes

No applicable. Although there were some minor technical issues encountered throughout the course of the study there were no significant issues or any major downtime affecting the delivery of the intervention or study results.

4a) CONSORT: Eligibility criteria for participants

Pg 6. "Participants were recruited from one VA Healthcare System between May 2009 and March 2011. Eligible participants were age 18 or older and identified through provider referrals to back class and use of the VA electronic medical record system. Specifically, we identified patients who had two or more outpatient encounters in the prior 12 months with a diagnosis of back pain with no neurologic findings (ICD-9-CM codes 724.2, 724.5, 846.0-846.9). Study staff screened potential participants by phone or in person. Eligibility criteria included: 1) persistent back pain > 3 months; 2) sedentary lifestyle (< 150 minutes of physical activity per week); 3) weekly access to a computer with a USB port and Internet access; 4) ability to provide written informed consent and communicate in English; 5) community residence; 6) ability to walk at least one block; and 7) report they are not pregnant. Prior to participation, all eligible patients had to attend back class and obtain medical clearance."

4a-i) Computer / Internet literacy

Pg 6. "Eligibility criteria included: 1) persistent back pain > 3 months; 2) sedentary lifestyle (< 150 minutes of physical activity per week); 3) weekly access to a computer with a USB port and Internet access;. . ."

4a-ii) Open vs. closed, web-based vs. face-to-face assessments:

Pg 6. "Participants were recruited from one VA Healthcare System between May 2009 and March 2011. Eligible participants were age 18 or older and identified through provider referrals to back class and use of the VA electronic medical record system. Specifically, we identified patients who had two or more outpatient encounters in the prior 12 months with a diagnosis of back pain with no neurologic findings (ICD-9-CM codes 724.2, 724.5, 846.0-846.9). Study staff screened potential participants by phone or in person."

Pgs 6-7. "Prior to participation, all eligible patients had to attend back class and obtain medical clearance. Back class, led by a physical therapist, provided general education about managing back pain. Participants also performed back specific strengthening and stretching exercises under the supervision of a physical therapist.
Eligible participants then attended a study enrollment session at which time they provided written informed consent and were told they were helping test an Internet-based program and would be assigned to one of two groups . . ."

4a-iii) Information giving during recruitment

Pg 7. "Eligible participants then attended a study enrollment session at which time they provided written informed consent and were told they were helping test an Internet-based program and would be assigned to one of two groups: 1) an enhanced care group that would upload pedometer data weekly and have access to a study website and computer discussion group (Internet support group); or 2) a usual care group that would upload pedometer data monthly (monthly upload group). All participants received an enhanced pedometer (the Omron HJ-720ITC, which stores 42 days of step-count data and has an embedded USB port [28]), along with general guidance on using the pedometer and instructions for logging onto and uploading data to the study website."

4b) CONSORT: Settings and locations where the data were collected

Pg 9. "Outcomes were measured at baseline, 6 months and 12 months using a survey administered through the study website, or by a mailed questionnaire if the participant could not complete the computerized instrument."

"Walking, both a secondary outcome and potential mediator, was measured as the average number of steps per day over the past 7 days using step-count data collected through the pedometer uploads."

4b-i) Report if outcomes were (self-)assessed through online questionnaires

Pg 9. "Outcomes were measured at baseline, 6 months and 12 months using a survey administered through the study website, or by a mailed questionnaire if the participant could not complete the computerized instrument."

"Walking, both a secondary outcome and potential mediator, was measured as the average number of steps per day over the past 7 days using step-count data collected through the pedometer uploads."

4b-ii) Report how institutional affiliations are displayed

Not discussed. The website included a link to view study sponsors. However, participants were Veterans receiving care through the Department of Veterans Affairs and were informed during recruitment/enrollment that the primary study sponsor was the Department of Veterans Affairs. Thus, we do not believe that how sponsorship information is displayed is particularly relevant for this study. However, the fact that this was a Veteran only population is acknowledged as a potential study limitation.

5) CONSORT: Describe the interventions for each group with sufficient details to allow replication, including how and when they were actually administered

5-i) Mention names, credential, affiliations of the developers, sponsors, and owners

Pg 8. "The study intervention, based on the Stepping Up to Health program developed by Richardson and colleagues . . ."

Additional detail about the developer and development of the program is provided in the cited references and published protocol paper.

5-ii) Describe the history/development process

Not applicable

Development described elsewhere, references provided.

5-iii) Revisions and updating

Pg 8. "The website also contained pain or activity-related motivational/informational messages, which changed every other day, and weekly updates about topics in the news. Back class materials and a video demonstrating the strengthening and stretching exercises also were available on the website. The e-community allowed participants and research staff to post suggestions, ask questions, share stories, and generate competitions to encourage meeting walking goals."

5-iv) Quality assurance methods

No applicable

5-v) Ensure replicability by publishing the source code, and/or providing screenshots/screen-capture video, and/or providing flowcharts of the algorithms used

Screenshots are included in a published protocol paper, which is included as one of the cited references.

5-vi) Digital preservation

Screen shots and program content have been archived. The program used for the intervention is no longer active.

5-vii) Access

The program used for the intervention is no longer active.

5-viii) Mode of delivery, features/functionalities/components of the intervention and comparator, and the theoretical framework

Pg 8. "The study intervention, based on the Stepping Up to Health program developed by Richardson and colleagues [29-30], consisted of three primary components: 1) the enhanced pedometer; 2) a website that provided automated goal setting and feedback, targeted messages and educational materials; and, 3) an e-community [27]. Intervention participants received weekly e-mail reminders to upload their pedometer data, weekly individualized walking goals and full access to the study website, which provided graphical and written feedback about their progress toward their walking goals. The website also contained pain or activity-related motivational/informational messages, which changed every other day, and weekly updates about topics in the news. Back class materials and a video demonstrating the strengthening and stretching exercises also were available on the website. The e-community allowed participants and research staff to post suggestions, ask questions, share stories, and generate competitions to encourage meeting walking goals.
Enhanced Usual Care
Usual care participants also received the enhanced pedometer and monthly e-mail reminders to upload their pedometer data. However, they did not receive any goals or feedback and their access to the study website was limited to completing surveys and reporting adverse events only."

The intervention components and the conceptual framework are discussed in further detail in a published protocol paper, which is included as a cited reference.

5-ix) Describe use parameters

Pg 7. "Eligible participants then attended a study enrollment session at which time they provided written informed consent and were told they were helping test an Internet-based program and would be assigned to one of two groups: 1) an enhanced care group that would upload pedometer data weekly and have access to a study website and computer discussion group (Internet support group); or 2) a usual care group that would upload pedometer data monthly (monthly upload group)."

5-x) Clarify the level of human involvement

Pg 6. "Study staff screened potential participants by phone or in person."

Pgs 6-7. "Prior to participation, all eligible patients had to attend back class and obtain medical clearance. Back class, led by a physical therapist, provided general education about managing back pain. Participants also performed back specific strengthening and stretching exercises under the supervision of a physical therapist."

Pg 7. "Eligible participants then attended a study enrollment session at which time they provided written informed consent and were told they were helping test an Internet-based program . . . ."

Pg 8. "The e-community allowed participants and research staff to post suggestions, ask questions, share stories, and generate competitions to encourage meeting walking goals."

Pgs 8-9. "Both groups were encouraged to report any health problems via the website, e-mail or phone. Four weeks after randomization and every eight weeks thereafter, participants were prompted to complete a survey that asked about specific adverse events (e.g., heart attack) and symptoms, such as shortness of breath. This information was closely monitored and participants with potentially serious health-related problems were contacted for further assessment and follow-up."

5-xi) Report any prompts/reminders used

Pg 8. "Intervention participants received weekly e-mail reminders to upload their pedometer data, weekly individualized walking goals and full access to the study website, which provided graphical and written feedback about their progress toward their walking goals."

"Usual care participants also received the enhanced pedometer and monthly e-mail reminders to upload their pedometer data."

"Both groups were encouraged to report any health problems via the website, e-mail or phone. Four weeks after randomization and every eight weeks thereafter, participants were prompted to complete a survey that asked about specific adverse events (e.g., heart attack) and symptoms, such as shortness of breath. "

5-xii) Describe any co-interventions (incl. training/support)

Pgs 6-7. "Prior to participation, all eligible patients had to attend back class and obtain medical clearance. Back class, led by a physical therapist, provided general education about managing back pain. Participants also performed back specific strengthening and stretching exercises under the supervision of a physical therapist."

6a) CONSORT: Completely defined pre-specified primary and secondary outcome measures, including how and when they were assessed

Pgs 9-10. "The pre-specified primary outcome was pain-related disability at 12 months, as measured using the back pain specific Roland and Morris Back Pain Disability Questionnaire (RDQ) [31], and a generic pain-related function measure from the medical outcomes study (MOS) [32]. The RDQ, a 24-item scale with higher scores indicating greater disability, has been widely used in back pain studies as a measure of self-perceived disability [31, 33-35]. The MOS measure assesses the effect of pain on mood and behaviors as well as pain severity, with higher scores also indicating greater functional interference [32].

Pain intensity, a secondary outcome, was evaluated using a numeric rating scale with standard anchors (0 = “no pain” and 10 = “worst pain imaginable”) [36]. Walking, both a secondary outcome and potential mediator, was measured as the average number of steps per day over the past 7 days using step-count data collected through the pedometer uploads. Other secondary outcomes and potential moderators included pain-related fear avoidance, measured using the Fear-Avoidance Beliefs Questionnaire physical activity sub-scale (higher scores reflect higher levels of fear-avoidance) [37], and self-efficacy for exercise, measured using the Exercise Regularly Scale, with higher scores indicating higher levels of self-efficacy [38]. Additional data collected at baseline included age, gender, race, employment status, education level, relationship status, average household income, body mass index, and use of narcotic medications for pain management. An administrative interface to the website provided data on the number of pedometer uploads and website logins."

6a-i) Online questionnaires: describe if they were validated for online use and apply CHERRIES items to describe how the questionnaires were designed/deployed

Not discussed. The specific questionnaires or measures were identified. All of the primary and secondary measures used previously validated instruments and measures. The questionnaire was pre-tested extensively using a paper version and on-line prior to beginning study enrollment.

6a-ii) Describe whether and how “use” (including intensity of use/dosage) was defined/measured/monitored

Pg 10. "An administrative interface to the website provided data on the number of pedometer uploads and website logins."

6a-iii) Describe whether, how, and when qualitative feedback from participants was obtained

Not discussed in this manuscript. Qualitative interviews were conducted with a few intervention participants, as described in the cited protocol paper. A quotation from one of these individuals is included in the discussion but the data were fairly limited and so considered as anecdotal and not suficient for a rigorous analysis.

6b) CONSORT: Any changes to trial outcomes after the trial commenced, with reasons

Pg 6. "There were no significant changes in methods following study initiation."

7a) CONSORT: How sample size was determined

7a-i) Describe whether and how expected attrition was taken into account when calculating the sample size

Pg 10. "Sample size was based on the RDQ score as the primary endpoint with a minimally detectable and clinically meaningful effect size determined as a difference of 0.4 standard deviations (SD) in change scores, or a 2 point difference at 12 months, based on published data [34, 39, 40]. To detect a difference of 0.4 SD with 80% power using a two-sided 0.05 level two-group t-test, we sought to enroll 130 subjects in each group, to allow for an attrition rate of 25% at one year."

7b) CONSORT: When applicable, explanation of any interim analyses and stopping guidelines

Both the interim (6 month) and final (12 month) results are presented.

8a) CONSORT: Method used to generate the random allocation sequence

Pg 7. "After completing the baseline survey, uploading seven days of useable pedometer data and receiving medical clearance, each participant was randomly allocated in a 1:1 ratio to the intervention or usual care group by a computer program (using a random number generator)."

8b) CONSORT: Type of randomisation; details of any restriction (such as blocking and block size)

Pg 7. "After completing the baseline survey, uploading seven days of useable pedometer data and receiving medical clearance, each participant was randomly allocated in a 1:1 ratio to the intervention or usual care group by a computer program (using a random number generator)."

9) CONSORT: Mechanism used to implement the random allocation sequence (such as sequentially numbered containers), describing any steps taken to conceal the sequence until interventions were assigned

Pg 7. "After completing the baseline survey, uploading seven days of useable pedometer data and receiving medical clearance, each participant was randomly allocated in a 1:1 ratio to the intervention or usual care group by a computer program (using a random number generator)."

10) CONSORT: Who generated the random allocation sequence, who enrolled participants, and who assigned participants to interventions

Pg 7. "After completing the baseline survey, uploading seven days of useable pedometer data and receiving medical clearance, each participant was randomly allocated in a 1:1 ratio to the intervention or usual care group by a computer program (using a random number generator)."

11a) CONSORT: Blinding - If done, who was blinded after assignment to interventions (for example, participants, care providers, those assessing outcomes) and how

11a-i) Specify who was blinded, and who wasn't

Pg 10. "The analyst assessing final trial outcomes was blinded to study assignment."

11a-ii) Discuss e.g., whether participants knew which intervention was the "intervention of interest" and which one was the "comparator"

Pg 7. "Eligible participants then attended a study enrollment session at which time they provided written informed consent and were told they were helping test an Internet-based program and would be assigned to one of two groups: 1) an enhanced care group that would upload pedometer data weekly and have access to a study website and computer discussion group (Internet support group); or 2) a usual care group that would upload pedometer data monthly (monthly upload group)."

11b) CONSORT: If relevant, description of the similarity of interventions

Pgs 6-7. "Prior to participation, all eligible patients had to attend back class and obtain medical clearance. Back class, led by a physical therapist, provided general education about managing back pain. Participants also performed back specific strengthening and stretching exercises under the supervision of a physical therapist."

Pg 7. "All participants received an enhanced pedometer (the Omron HJ-720ITC, which stores 42 days of step-count data and has an embedded USB port [28]), along with general guidance on using the pedometer and instructions for logging onto and uploading data to the study website. To collect baseline step-count data, participants were instructed to wear their pedometer for seven days with the display covered before completing their first upload."

12a) CONSORT: Statistical methods used to compare groups for primary and secondary outcomes

Pgs 10-11. "All analyses were conducted using an intent-to-treat approach with participants analyzed according to original group assignment. We conducted both complete and all case analyses to assess differences between groups in change in RDQ at 6 and 12 months. The complete case analysis was conducted using multiple linear regression models with adjustment for baseline values of the RDQ. The all case analysis was conducted using linear mixed-effects models, allowing us to use data from all participants and provide an unbiased estimate of the outcome, assuming data are missing at random. For example, for our 12-month analysis, RDQ scores at baseline and 12 months were used as dependent variables, while the independent variables consisted of baseline RDQ values, an indicator for the intervention group and an interaction term of time by intervention group. Each participant's data was modeled using a random intercept to allow within-patient correlation of the repeated measures. Adjustment for covariates was only planned if an imbalance was found between groups at baseline."

12a-i) Imputation techniques to deal with attrition / missing values

Pgs 10-11. "We conducted both complete and all case analyses to assess differences between groups in change in RDQ at 6 and 12 months. The complete case analysis was conducted using multiple linear regression models with adjustment for baseline values of the RDQ. The all case analysis was conducted using linear mixed-effects models, allowing us to use data from all participants and provide an unbiased estimate of the outcome, assuming data are missing at random. For example, for our 12-month analysis, RDQ scores at baseline and 12 months were used as dependent variables, while the independent variables consisted of baseline RDQ values, an indicator for the intervention group and an interaction term of time by intervention group. Each participant's data was modeled using a random intercept to allow within-patient correlation of the repeated measures. Adjustment for covariates was only planned if an imbalance was found between groups at baseline."

12b) CONSORT: Methods for additional analyses, such as subgroup analyses and adjusted analyses

Pg 11. "Adjustment for covariates was only planned if an imbalance was found between groups at baseline."

"We also conducted a post-hoc subgroup analysis of participants with baseline RDQ scores of ≥ 4. As a pragmatic trial we did not screen based on RDQ scores, and some participants had baseline scores that were very low or even zero. Thus, to assess the effect of the intervention on participants reporting at least modest levels of back pain related disability at baseline we conducted a subgroup analysis of those with baseline RDQ scores of ≥ 4 using the same methods previously described."

RESULTS

13a) CONSORT: For each group, the numbers of participants who were randomly assigned, received intended treatment, and were analysed for the primary outcome

Pg 11. "Over 1400 potential participants (Figure 1) were assessed for eligibility. Primary reasons for ineligibility were lack of regular access to a computer or the Internet (n = 310) and being too physically active (n = 159). Of those determined to be eligible, 229 completed all of the steps in the enrollment process, with 111 randomly allocated to the Internet-mediated intervention and 118 to enhanced usual care. Primary outcome data were provided by 91% of intervention and 90% of usual care participants at 6 months, and by 92% of those in the intervention group and 89% receiving usual care at 12 months."

CONSORT flow diagram also included as Figure 1.

13b) CONSORT: For each group, losses and exclusions after randomisation, together with reasons

Pg. 12, CONSORT flow diagram included as Figure 1

13b-i) Attrition diagram

Pg 18. "Intervention participants uploaded pedometer data at least once per week for a median of 32 weeks (62% of the recommended time) although more than 25% of participants uploaded data at least 42 weeks (80% compliance). However, intervention participants logged into the website at least once per week for a median of only 20 weeks (38% of the recommended time), with approximately 20% logging in at least 42 weeks."

14a) CONSORT: Dates defining the periods of recruitment and follow-up

Pg 6. "Participants were recruited from one VA Healthcare System between May 2009 and March 2011."

Pg 9. "Outcomes were measured at baseline, 6 months and 12 months . . ."

14a-i) Indicate if critical "secular events" fell into the study period

No critical events identified during the study timeframe

14b) CONSORT: Why the trial ended or was stopped (early)

Not discussed. The trial ended after all recruited participants had been followed for 12 months.

15) CONSORT: A table showing baseline demographic and clinical characteristics for each group

Pgs. 13-14, Table 1 with a description of baseline characteristics for both groups

15-i) Report demographics associated with digital divide issues

Pgs. 13-14, Table 1 with a description of baseline characteristics for both groups includes some of these characteristics.

Computer and Internet use/access were included as part of eligibility criteria.

16a) CONSORT: For each group, number of participants (denominator) included in each analysis and whether the analysis was by original assigned groups

16-i) Report multiple “denominators” and provide definitions

Results shown in table 2 and 3 specify whether complete or all case analysis, which takes into account missing data. For the complete case analysis, the denominators are generally consistent with the number of participants who provided data at each time point as noted in Figure 1 with one exception, which is the pedometer step count data as noted in the footnote to Table 3.

16-ii) Primary analysis should be intent-to-treat

Pg 10. "All analyses were conducted using an intent-to-treat approach with participants analyzed according to original group assignment."

17a) CONSORT: For each primary and secondary outcome, results for each group, and the estimated effect size and its precision (such as 95% confidence interval)

Presented in Tables 2 and 3

17a-i) Presentation of process outcomes such as metrics of use and intensity of use

Pg 18. "Intervention participants uploaded pedometer data at least once per week for a median of 32 weeks (62% of the recommended time) although more than 25% of participants uploaded data at least 42 weeks (80% compliance). However, intervention participants logged into the website at least once per week for a median of only 20 weeks (38% of the recommended time), with approximately 20% logging in at least 42 weeks."

17b) CONSORT: For binary outcomes, presentation of both absolute and relative effect sizes is recommended

Not applicable. Outcomes are continuous

18) CONSORT: Results of any other analyses performed, including subgroup analyses and adjusted analyses, distinguishing pre-specified from exploratory

Pgs. 14-15. "When restricted to the subgroup with at least moderate back pain at baseline (RDQ score ≥ 4) (Figure 2, Table 2), patients in the intervention had a significant improvement in back pain-related disability compared to the control group, an adjusted difference of approximately 2 in both the complete (1.9, 95% CI: 0.5-3.3, P = .01) and all case (1.7, 95% CI: 0.3-3.0, P = .02) analyses. RDQ scores continued to decline between 6 and 12 months in both groups and while scores for the intervention group remained lower than for usual care, at 12 months these differences were no longer statistically significant."

Table 1 also presents subgroup analysis as well as full group analysis for primary outcomes

18-i) Subgroup analysis of comparing only users

Not applicable

19) CONSORT: All important harms or unintended effects in each group

Pg 19. "During the study, approximately 600 adverse events were reported by participants (250 by those in usual care and nearly 350 by those in the intervention). These events ranged from calluses to chest pain. Worsening back pain, the most frequently reported event, accounted for 29% of events reported by the usual care group and 25% of those reported by the intervention group. Overall, more musculoskeletal events (n = 112) were reported than cardiovascular events (n = 85) and musculoskeletal injuries were more likely to be reported by participants in the intervention group compared to those in usual care. However, no major study-related adverse events (e.g., heart attack) were identified for either group."

19-i) Include privacy breaches, technical problems

Not applicable, no specific problems identified.

19-ii) Include qualitative feedback from participants or observations from staff/researchers

Pg 20. "As one intervention participant noted: “I didn’t know what the walking could do for me. But. . . it seemed to alleviate my back pain . . . the true test came when I had to go off the program because of my illness and the back pain returned. In fact, just up until recently, when I had resumed walking.”"

Quote from participant included in discussion as anecdotal. Qualitative data were collected through semi-structured interviews with a few intervention participants but was realtively limited and not amenable to a systematic or rigorous analysis process.

DISCUSSION

20) CONSORT: Trial limitations, addressing sources of potential bias, imprecision, multiplicity of analyses

20-i) Typical limitations in ehealth trials

Pg 21. "This study also has several limitations. First, patients were recruited from only one medical center and the sample was predominantly male. Although over 10% of participants were female, which is relatively high for studies using a general VA patient population, the number is not sufficient for a formal subgroup analysis. However, based on trials of similar types of interventions we expect this approach could be even more effective among women [50]. Second, we are not able to directly compare our results to other types of back pain interventions (e.g., yoga), although as previously noted the general trajectory of our primary outcome (RDQ score) appears consistent with recent trials in this area. Finally, as a multi-faceted intervention we are not able to determine which elements were most effective and can only draw conclusions about the program as a whole. Nonetheless, our results highlight the importance of providing active support (e.g., goal setting and feedback) to encourage walking as compared with simply giving someone a pedometer to track step counts."

21) CONSORT: Generalisability (external validity, applicability) of the trial findings

21-i) Generalizability to other populations

Pg 21. "This study also has several limitations. First, patients were recruited from only one medical center and the sample was predominantly male. Although over 10% of participants were female, which is relatively high for studies using a general VA patient population, the number is not sufficient for a formal subgroup analysis. However, based on trials of similar types of interventions we expect this approach could be even more effective among women [50]."

21-ii) Discuss if there were elements in the RCT that would be different in a routine application setting

Not explicitly discussed, although the potential need for additional support to facilitate ongoing use as part of routine care are described as an avenue for further research.

22) CONSORT: Interpretation consistent with results, balancing benefits and harms, and considering other relevant evidence

22-i) Restate study questions and summarize the answers suggested by the data, starting with primary outcomes and process outcomes (use)

Pg 19. "Our findings show that an automated, Internet-mediated walking intervention may help to reduce back-pain related disability among patients with chronic back pain, although the benefits did not persist for the entire 12-month study period. Improvement was greatest for those individuals reporting moderate to severe levels of pain-related disability at baseline."

22-ii) Highlight unanswered new questions, suggest future research

Pgs 19-20. " . . .even though we did not observe additional improvements in function at 12 months, our findings suggest that automated, remotely delivered interventions can be effectively used to encourage physical activity and supplement care for patients with chronic low back pain. Further investigation is needed, however, to understand the characteristics of patients who had an early or enduring response to the intervention so that we may better target patients most likely to benefit and broaden the response."

Pg 20. "However, because step count improvements were not sustained for the entire 12 months, additional strategies to keep people active also may be needed. This could include, for example, an online coaching component, which has been shown to improve adherence to other types of behavioral changes [47-49]."

Other information

23) CONSORT: Registration number and name of trial registry

Pg 4. ClinicalTrials.gov NCT00694018

24) CONSORT: Where the full trial protocol can be accessed, if available

A published protocol paper is included in the cited references and the full protocol is available upon request.

25) CONSORT: Sources of funding and other support (such as supply of drugs), role of funders

Pg 22. Included in acknowledgments: "This project was funded through a grant from the Department of Veterans Affairs, Health Services Research and Development Service (IIR 07-177). The views expressed in this article are those of the authors and do not necessarily reflect the position or policy of the Department of Veterans Affairs. The study sponsors had no role in the design or conduct of the study; the collection, management, analysis and interpretation of the data; or the preparation, review or approval of the manuscript. Dr. Piette is a VA Senior Research Career Scientist. Dr. Richardson was supported in part by a career development award from NHLBI (K23HL075098) and a Physician Faculty Scholars Program award from the Robert Wood Johnson Foundation (57408). Development of the Stepping Up to Health intervention platform was supported by pilot grant funding from the following University of Michigan centers: Michigan Diabetes Research and Training Center (P60 DK020572), the Center for Health Communications Research (P50 CA101451), the Michigan Institute for Clinical and Health Research (NIH #UL1RR024986)."

X26-i) Comment on ethics committee approval

Pg 6. "This research was approved by the Department of Veterans Affairs (VA) Ann Arbor Healthcare System institutional review board."

x26-ii) Outline informed consent procedures

Pg 7. "Eligible participants then attended a study enrollment session at which time they provided written informed consent and were told they were helping test an Internet-based program and would be assigned to one of two groups . . ."

X26-iii) Safety and security procedures

Pg 8. "Both groups were encouraged to report any health problems via the website, e-mail or phone. Four weeks after randomization and every eight weeks thereafter, participants were prompted to complete a survey that asked about specific adverse events (e.g., heart attack) and symptoms, such as shortness of breath. This information was closely monitored and participants with potentially serious health-related problems were contacted for further assessment and follow-up."

X27-i) State the relation of the study team towards the system being evaluated

Pg 8. "The study intervention, based on the Stepping Up to Health program developed by Richardson and colleagues [29-30], . . ."

Pg 22. In acknowledgments. "Development of the Stepping Up to Health intervention platform was supported by pilot grant funding from the following University of Michigan centers: Michigan Diabetes Research and Training Center (P60 DK020572), the Center for Health Communications Research (P50 CA101451), the Michigan Institute for Clinical and Health Research (NIH #UL1RR024986). "
